# Covalent Attachment of Proteins to Solid Supports and Surfaces via Sortase-Mediated Ligation

**DOI:** 10.1371/journal.pone.0001164

**Published:** 2007-11-14

**Authors:** Lilyan Chan, Hannah F. Cross, Joseph K. She, Gabriel Cavalli, Hugo F. P. Martins, Cameron Neylon

**Affiliations:** 1 School of Chemistry, University of Southampton, Highfield, Southampton, United Kingdom; 2 School of Biomedical and Molecular Sciences, University of Surrey, Guildford, United Kingdom; 3 Science and Technology Facilities Council Rutherford Appleton Laboratory, Harwell Science and Innovation Campus, Didcot, United Kingdom; Massachusetts Institute of Technology, United States of America

## Abstract

**Background:**

There is growing interest in the attachment of proteins to solid supports for the development of supported catalysts, affinity matrices, and micro devices as well as for the development of planar and bead based protein arrays for multiplexed assays of protein concentration, interactions, and activity. A critical requirement for these applications is the generation of a stable linkage between the solid support and the immobilized, but still functional, protein.

**Methodology:**

Solid supports including crosslinked polymer beads, beaded agarose, and planar glass surfaces, were modified to present an oligoglycine motif to solution. A range of proteins were ligated to the various surfaces using the Sortase A enzyme of *S. aureus*. Reactions were carried out in aqueous buffer conditions at room temperature for times between one and twelve hours.

**Conclusions:**

The Sortase A transpeptidase of *S. aureus* provides a general, robust, and gentle approach to the selective covalent immobilization of proteins on three very different solid supports. The proteins remain functional and accessible to solution. Sortase mediated ligation is therefore a straightforward methodology for the preparation of solid supported enzymes and bead based assays, as well as the modification of planar surfaces for microanalytical devices and protein arrays.

## Introduction

A wide range of protein-based technological applications require a linkage to be formed between a protein and a solid support. Solid supported enzymes offer a means of recovering and re-using costly protein catalysts. A range of modern assay approaches require the attachment of specific proteins to specific encoded beads [1], [2] or microparticles [3]. The development of microfluidic analytical devices is based in a number of cases on a probe protein attached to surfaces within the microdevice [4] or to beads placed within a device [5]. Finally the development of planar protein arrays requires that each protein is immobilised within a defined area on the array surface.

A range of immobilisation approaches are available including simple absorption, trapping the protein within a gel matrix, or covalent linkage. Absorption is often used in microplate immunoassays as a simple method for immobilisation that provides sufficient active antibody for the assays. However absorption poses two risks: that the protein may denature on the surface or that it may wash off under assay or storage conditions. Trapping proteins within a gel matrix has been applied to the generation of protein arrays with significant success. This provides a gentle immobilisation approach that usually maintains protein activity.

The most robust approach to protein immobilisation is through a covalent linkage. Non-specific covalent linkage through sidechain amino groups to activated surface functional groups [6] works well for robust proteins with relaxed orientation requirements such as antibodies but in situations where surface homogeneity and orientation is important these methods may not be appropriate [7]. Site specific reaction of existing or engineered cysteine residues with activated or gold surfaces can provide selective and oriented attachment [8], [9] but are limited to situations where cysteine residues can be optimally positioned and/or removed from the native protein sequence.

In recent years a number of selective ligation chemistries have been developed that can be applied to biological systems. Both Staudinger ligation and ‘Click’ chemistry generate covalent linkages from highly selective reactions of functional groups that are generally not found in biological systems. In both cases the incorporation of an azide into the protein to be modified is required. This can be achieved through misincorporation of azido functionalised amino acids in auxotrophic strains [10], through chemical modification of functional groups in amino acids within the native or modified protein sequence [11], [12], or through the selective modification of protein prosthetic groups [13]. The first two approaches for azide incorporation will in general be non-specific and providing a single point of attachment will require further manipulation of the protein sequence in many cases. Modification of prosthetic groups [13] will be convenient in some instances but will be limited to particular cases.

Immobilization via expressed protein ligation has been successfully applied to a number of systems both directly [14], [15] and via the addition of affinity reagents biotin [16] or specific functional groups including azides [17], [18]. The use of intein-based systems provides a site-selective method but is often limited due to difficulties with expression of large target-intein fusion proteins and side reactions of the thiol based chemistry. In addition many of these modification approaches require multiple manipulations steps, cloning and modification of the protein encoding gene, expression and incorporation of the affinity label or functional group, and finally attachment to the solid support. In addition the use of intein based methods as well as the preparation of the solid support for Staudinger ligation often require reagents such as phosphines or thiophenols that are toxic and difficult to handle.

Therefore there remains a significant need for robust and simple methodologies for protein immobilization that can be applied to wide range of proteins and solid supports. The identification of the Sortase transpeptidase [19] provided an alternative approach to protein ligation. Sortases recognise a specific peptide sequence (LPETG for SrtA of *S. aureus* used in this work) in proteins targeted for covalent attachment to the cell wall peptidoglycan. The peptide tag sequence is cleaved and then ligated to the pentaglycine moiety on the peptidoglycan precursor Lipid II. Proteins expressed with the C-terminal recognition sequence can be covalently attached to a wide range of constructs with an N-terminal glycine amide motif including peptides [20], PNA [21], full length proteins [22] and small molecule substrates [23]. Another group has independently described an example of Sortase mediated ligation to a beaded solid support [22]. These reactions proceed under aqueous conditions without the addition of any further reagents beyond the protein, ligation substrate, and Sortase. Thus Sortase has the potential to provide a means of linking expressed proteins to a wide range of solid supports which is mild, selective, and can be carried out in a single step. Here we investigate the ability of *S. aureus* SrtA to ligate proteins to a range of solid supports.

## Results

Plasmid vectors were constructed for the expression of Blue fluorescent protein (BFP, Q-Biogene), Enhanced Green Fluorescent Protein (EGFP), a red fluorescent protein (DsRed), and the sequence specific DNA binding protein Tus [24] with a C-terminal LPETGG sequence followed by a hexahistidine tag. The proteins were expressed in BL21(DE3) and purified before attachment to solid supports.

**Figure 1:**
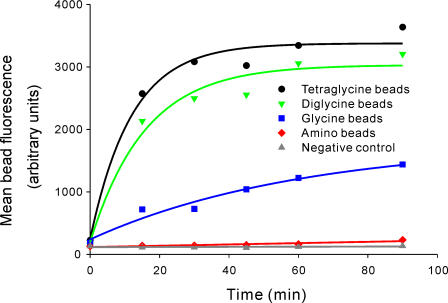
Ligation of fluorescent proteins to polymer beads. (a) GMA beads modified with one, two, or four glycine residues were incubated with EGFP-LPETGG-His_6_ and Sortase. Samples were taken at specific time points and analyzed on a BD FACSAria. Controls contained beads with no glycine or diglycine beads without Sortase. Error bars showing the standard error in the mean fluorescence are omitted as they are generally smaller than the data symbols. Errors are given in Supplementary Data S2.

**Figure 2:**
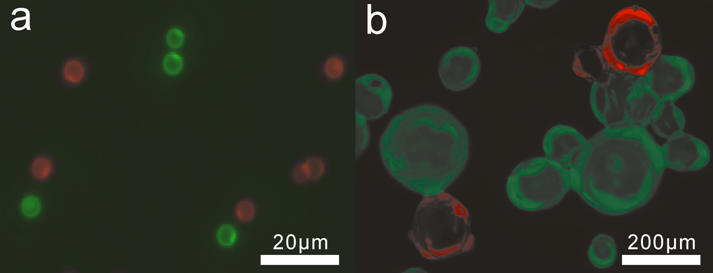
Fluorescence micrographs of labeled solid supports. (a) Diglycine GMA beads and (b) oligoglycine modified Affigel resin were separately labeled with EGFP and DsRed and then mixed. Fluorescence images were recorded as separate gray scale images (see Supplementary Figure S2) with FITC and Cy3 filter sets and then combined and false coloured.

Our first target was the immobilization of proteins onto cross-linked polymer beads. Glycidyl methacrylate (GMA) beads were modified with a spacer followed by one, two, or four glycine residues. Mono-glycine, di-glycine, and tetra-glycine beads were incubated with EGFP-LPETGG-His_6_ (85 µM) and His_6_-Sortase A (40 nM) in Sortase buffer (50 mM Tris-HCl, 150 mM NaCl, 5 mM CaCl_2_, pH 7.5). As a control, beads with no glycine coupled were incubated with Sortase and EGFP-LPETGG-His_6_, and tetraglycine beads were incubated with EGFP-LPETGG-His_6_ in the absence of Sortase. Samples were taken at various time points and beads analysed by FACS (Figure 1) and fluorescence microscopy (Figure 2). The labeled beads were clearly visible by fluorescence microscopy while beads from control reactions showed no increase in fluorescence. Tetra-glycine beads showed the most rapid fluorescence increase and the highest final fluorescence. Di-glycine beads were nearly as effective as tetra-glycine with mono-glycine beads showing slower increase and significantly reduced final fluorescence.

**Figure 3:**
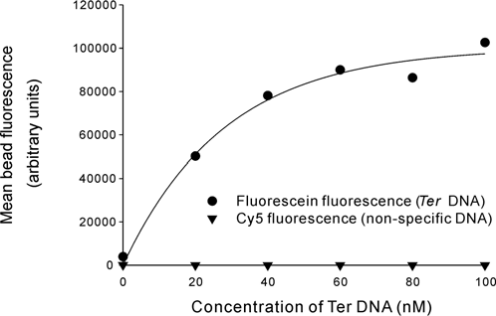
Tus protein ligated to GMA beads is accessible to its cognate DNA ligand (*Ter*). The sequence specific DNA-binding protein Tus was ligated to diglycine GMA beads. The Tus-labeled beads were incubated with varying proportions of fluorescein labeled *Ter* DNA and Cy5 labeled non-specific DNA and the bead fluorescence analysed by FACS. The curve is a model fit for a single binding process with a *K*_D_ of 29±8 nM. Error bars showing the standard error in the mean fluorescence are omitted as they are generally smaller than the data symbols. Errors are given in Supplementary Data S2.

EGFP and the other fluorescent proteins are extremely robust. While the fluorescence analysis of the ligation of these proteins demonstrates maintenance of function it is therefore of interest to demonstrate that more fragile proteins can be ligated to solid supports while maintaining function. This is crucial for applications in supported catalysis or bead-based protein arrays. To demonstrate that ligated protein was functional and accessible to other molecules in solution Tus-LPETGG-His_6_ was immobilized on tetraglycine GMA beads. Tus is a sequence specific DNA-binding protein that recognizes 21 bp *Ter* sites [24]. The Tus-labeled GMA beads were incubated with different proportions of fluorescein labeled *TerB* DNA and a 21 bp Cy5-labeled DNA sequence unrelated to *TerB* in binding buffer (50 mM Tris-HCl, 250 mM KCl, 0.1 mM EDTA, 0.1 mM DTT, pH 9). The total DNA concentration (*Ter* plus nonspecific DNA) was 100 nM for all samples. The fluorescein and Cy5 fluorescence of the beads was determined by FACS analysis. Non-specific DNA binding was very low in all cases, consistent with the low affinity of Tus for non-specific DNA in 250 mM KCl [25]. *Ter* binding showed a concentration dependence that was consistent with an equilibrium dissociation constant of 29±8 nM (Figure 3), which compares well with values of *K*_D_ measured by fluorescence anisotropy (∼15 nM at 37°C) or Biacore (∼1 nM at 25°C) [25].

**Figure 4:**
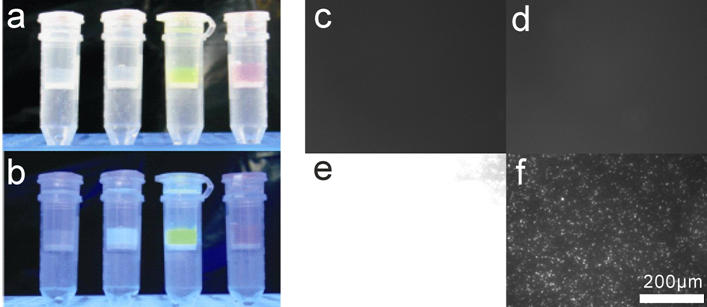
(a,b) Ligation of fluorescent proteins to Affi-Gel resin. Left to right, negative control, BFP-, EGFP-, and DsRed-LPETGG-His_6_ were ligated to Affi-Gel 102 Resin modified with oligoglycine. The negative control reaction contained EGFP-LPETGG-His_6_ and no Sortase. After washing with buffer pictures were taken with a white light (a) and UV transilluminator (b, 312 nm). (c–f) Ligation of EGFP to a glass surface. Microscope coverslips were modified with triethoxy(aminopropyl) silane and oligoglycine before incubation with EGFP-LPETGG-His_6_. The slides were washed with Sortase buffer containing 1% SDS and photographed using the FITC filter set on an Axiovert 200 microscope. (c) Glycine modified surface with EGFP-LPETGG-His_6_ but no Sortase, (d) amino modified surface (i.e. without glycine) with EGFP-LPETGG-His_6_ and Sortase, (e) Glycine modified surface with EGFP-LPETGG-His_6_ and Sortase with same exposure settings as negative controls, (f) same as (e) with five-fold reduced exposure time.

To demonstrate attachment to other solid supports a beaded agarose affinity support (Affi-Gel 102 resin, Bio-Rad) was modified with oligoglycine by incubating the amino-resin with diglycine (0.5 M) and EDC (2.5 mM) for three hours at 50°C. BFP-, EGFP-, and DsRed-LPETGG-His_6_ were then ligated to the resin overnight at room temperature. After washing the resin columns could be seen to be clearly labeled with fluorescent protein (Figure 4a,b) whereas a control (EGFP without Sortase) showed no fluorescence. The labeled Affi-Gel beads were clearly visible by fluorescence microscopy (Figure 2).

Finally we sought to demonstrate ligation to surfaces applicable to the development of planar protein arrays or microdevices. Clean glass coverslips were modified with an aminosilane and oligoglycine was then coupled to the amino-modified surface. EGFP-LPETGG-His_6_ was then ligated overnight to the slide at room temperature. In the absence of Sortase or of oligoglycine no fluorescence was observed (Figure 4c,d). In a separate experiment no fluorescence was observed for ligation of EGFP without the LPETGG tag in the presence of Sortase. A high fluorescence was observed for the positive slide. The labeling is continuous across the modified surface with spots of higher fluorescence (Figure 4e,f).

## Discussion

Protein ligation to solid supports and surfaces can be a challenging problem. For the examples investigated here Sortase-mediated ligation has provided a very straightforward and effective means of achieving ligation both to bead based solid supports and to surfaces. The ligation provides selective and oriented attachment and can be carried out at room temperature under mild aqueous conditions and without apparent loss of protein activity or specificity. We have not observed any problems with non-specific binding of proteins under the ligation conditions used here.

An important parameter for optimisation of ligation, especially enzymatic ligation, is the spacer provided between the surface of the solid support and the point of attachment. In addition to the issue of space there is the question of how many glycine residues are required for optimal attachment by Sortase. On GMA beads we observe a clear relationship between the number of glycine residues and the rate and yield of attachment with four glycine residues giving the highest yield and most rapid reaction. Two glycine residues provides nearly as effective attachment with several fewer support modification steps required making this the most likely choice for practical applications. The linker dependence contrasts with previously reported studies of the substrate specificity of Sortase where increasing the number of glycines in small molecule substrates did not lead to an increase in activity or substrate binding beyond two glycines and indeed glycinamide was nearly as effective a substrate as diglycine [26]. In preliminary experiments where we attempted to ligate EGFP to planar gold surfaces through immobilised Gly_2_-, Gly_3_-, or Gly_4_-Cys peptides we were unable to observe any increase in fluorescence above background (see Supplementary Figure S3 for an example). These results taken together suggest a complex interaction between the substrate specificity of Sortase and problems of steric hindrance. The random coupling of oligoglycine provides a convenient means of overcoming this apparent steric hindrance but the current data does not provide sufficient evidence to identify the optimum linker length for planar surfaces or Affigel. The details of linker lengths and identify for the optimised use of Sortase mediated ligation both to solid supports and to other molecules will need to be established by future work.

In comparing Sortase mediated ligation to other methods for the conjugation of proteins to solid supports it is important to emphasise the ease of use of this method. Sortase provides a means of directly ligating expressed protein with no further modification required to solid supports. Appropriate supports can be readily prepared using straightforward chemistry that is accessible to workers from a wide range of scientific and technical disciplines. There is no requirement for modification of the internal protein sequence by removing, for example, undesired cysteines. Nor is there any requirement for modification of the protein at the chemical level by misincorporation of unnatural amino acids or by further modification of the expressed protein. There is additionally no requirement to handle potentially toxic reagents such as phosphines in the preparation of the solid supports. The major drawback of Sortase mediated ligation is that in its current form it is limited to ligation through the C-terminus of the protein. If the C-terminus of a protein is required for function then this will not be an appropriate method. The second potential drawback of Sortase mediated ligation is the requirement for the preparation of Sortase. However the protein is easily over expressed and purified in large yields.

In conclusion we have shown that Sortase-mediated ligation provides a convenient and mild approach to the covalent linkage of proteins to a range of relevant solid supports including cross-linked polymer beads, affinity resins, and flat surfaces. This approach is applicable to any protein that can be functionally expressed and purified with a short peptide tag at the C-terminus of the protein. This makes the approach widely applicable to proteins which have specific orientational requirements or which are rendered inactive by non-specific modification. In contrast to the use of target-intein fusions for immobilisation, where the expression of large fusion proteins can be a problem, the small tag is much less likely to have an effect on expression. In addition Sortase-mediated ligation is applicable to any solid support which can be modified to provide an oligoglycine motif. Sortase therefore provides a robust method for protein ligation to solid supports that is effective, easy to use, and very general.

## Materials and Methods

### Plasmids and protein expression

The *srtA* gene was amplified from *S. aureus* genomic DNA (ATCC 35556D) using primers *srtaf* (5′-ATATTTGCATATGAAACCACATATCGATAATTATC) and *srtab* (5′-ATTGAATTCGATTATTTGACTTCTGTAGCTACAAG) which remove the N-terminal membrane targeting region and add an *Nde*I site at the start codon and add an *Eco*RI site just beyond the stop codon (restriction sites underlined). The PCR product was digested with *Nde*I and *Eco*RI and inserted into pETMCSIII [25] for the expression of a SortaseA construct with the N-terminal membrane targeting sequence removed and replaced with a His_6_ tag for affinity purification, similar to that previously described [19]. The protein was readily purified in high yield by Ni-affinity chromatography. For reasons which are not clear Sortase migrates anomalously in SDS-PAGE with an apparent molecular weight of ∼30 kDa (see also Figure 5 in Reference [19]).

Plasmid vectors for the expression of target proteins with a C-terminal LPETGG-His_6_ tag were designed with an in frame *Xho*I site to replace the native stop codon and an *Nde*I site at the start codon. Genes coding for BFP (derived from pQBI-T7-BFP, Q-Biogene, http://www.qbiogene.com/technical/maps/txt/s-pQBI-T7-BFP.txt), EGFP (BFP with S66T, H67Y mutations), DsRed (derived from pDsRed-Monomer, Clontech, http://www.clontech.com/images/pt/PT3794-5.pdf), and the *E. coli* Tus protein (derived from pCM862 [25]) were inserted into these vectors, and protein expressed and purified by Ni-affinity chromatography (BFP-, EGFP-, DsRed-LPETGG-His_6_) or a two column ion exchange procedure adapted from that previously described (Tus-LPETGG-His_6_ [25]). The details of the plasmid construction and protein purification will be reported elsewhere. The full predicted sequence of the proteins is given in Supplementary Data S1.

### Manipulations of solid supports

GMA beads (5 µm, Bangs Laboratories) were modified with an aminooctanoic acid spacer and one, two, or four glycine residues added by standard Fmoc chemistry with TBTU coupling. The final loading of the beads (by quantitative ninhydrin test) was 5 µmol.g^−1^ for all but tetra-glycine beads (2 µmol.g^−1^).

Affi-Gel 102 Resin (Bio-Rad) was modified with oligoglycine by incubation with diglycine (0.5 M) and EDC (2.5 mM) for three hours at 50°C. Clean glass coverslips were incubated in 2% triethoxy(aminopropyl) silane for 30 seconds, washed with acetone and air dried. Oligoglycine was coupled to the surface overnight by incubation with diglycine (0.5 M) and EDC (2.5 mM) at room temperature.

### Collection of FACS data

FACS data was obtained on a BD FACSAria. Beads were collected by centrifugation and resuspended in FacsFlow (BD Bioscience). A total of 1000 events (Figure 1) or 5000 events (Figure 3) was recorded for each time point. The population of single beads (i.e. not bead doublets or higher aggregates) was selected based on forward and side scatter) and the mean fluorescence of this population determined (Fluorescein: 488 nm laser excitation, 530/30 nm emission filter, Cy5: 633 nm laser excitation, 670/20 emission filter, see Supplementary Data S2 for data used to generate Figures 1 and 3 and Supplementary Figure S1 for an example of the raw FACS data).

### Fluorescence microscopy

Images were obtained on a Zeiss Axiovert 200 fluorescence microscope with mercury vapour lamp illumination and FITC and Cy3 filter sets. Images were captured on a Hamamatsu ORCA-ER grey scale digital camera using Simple PCI software. For colour panels in Figure 2 separate grey scale images were taken with FITC and Cy3 filter sets with the autoexposure setting in Simple PCI. The grey scale images were combined and false colored using Corel Draw. The original grey scale images are available in Supplementary Figure S2. The images in Figure 4 c,d,e were taken with a gain of 255 and an exposure of 0.0125 seconds. Figure 4f was taken with an exposure time of 0.0025 seconds.

## Supporting Information

Data S1 Predicted amino acid sequences for the proteins used in this work.
(0.03 MB DOC)

Data S2 FACS data used to prepare Figure 1 and Figure 3. The mean fluorescence and standard error in the mean are given along with the number of events contributing to those values.
(0.05 MB DOC)

Figure S1 Example raw FACS data for the labeling of diglycine modified GMA beads with EGFP-LPETGG-His6. A) Forward scatter versus side scatter (left panel). The central population (P1) consisting of single isolated beads is selected for further analysis. The values reported in Figure 1 are derived from analysis of this population. Forward scatter versus EGFP fluorescence (right panel) for diglycine beads after 30 minutes incubation with EGFP-LPETGG-His6 and Sortase. B) Histograms showing the increase in fluorescence over the course of the incubation. Times are as given at the top of each panel. The green population is the P2 selected in the the right hand panel of A). In all cases it is almost perfectly overlayed over the red P1 population selected on the basis of forward and side scatter, and from which average fluorescences were calculated.
(0.04 MB DOC)

Figure S2 Original grey scale images used to construct Figure 2. Top panel show originals for Figure 2a and bottom panels show the original images used for preparation of Figure 2b. The dashed box shows the area of the original image that was expanded. Grey scale images were taken using the FITC or Cy3 filter sets on a Zeiss Axiovert 200 microscope using mercury vapour lamp illumination and captured on a Hamamatsu ORCA-ER camera using Simple PCI software. The exposure settings were selected using the ‘Auto Exposure’ setting of Simple PCI for each image.
(1.53 MB DOC)

Figure S3 Unsuccessful preliminary attempts to immobilise EGFP on planar gold surfaces. The peptide H-GGC-OH (1 mM) was incubated overnight with a gold coated glass slide in the presence of triscarboxyethylphosphine (TCEP). The ligation of EGFP was attempted in the presence of 50 µM EGFP and 40 nM Sortase overnight. The successful immobilisation of the glycine peptide was not confirmed. Upper panel negative control (no Sortase). Lower panel, attempted ligation with Sortase. The lower panel is slightly out of focus. Similar results were seen with the peptides H-G3C-OH and H-G4C-OH.
(0.53 MB DOC)
