# Supplementary material for: Covalent Attachment of Proteins to Solid Supports and Surfaces via Sortase-Mediated Ligation
Source: PLoS One. 2007 Nov 14;2(11):e1164. doi: 10.1371/journal.pone.0001164 (PMC2063460; doi:10.1371/journal.pone.0001164)
Supplement: Data S1 — Predicted amino acid sequences for the proteins used in this work. (0.03 MB DOC) [file pone.0001164.s001.doc]

**Supplementary Data S1**

**Predicted amino acid sequences of proteins used in this work.**

BFP-LPETGG-His_6_

MASKGEELFTGVVPILVELDGDVNGHKFSVSGEGEGDATYGKLTLKFICT

TGKLPVPWPTLVTTLSHGVQCFSRYPDHMKRHDFFKSAMPEGYVQERTIF

FKDDGNYKTRAEVKFEGDTLVNRIELKGIDFKEDGNILGHKLEYNYNSHN

VYIMADKQKNGIKANFKIRHNIEDGSVQLADHYQQNTPIGDGPVLLPDNH

YLSTQSALSKDPNEKRDHMVLLEFVTAAGITHGMDELYKGSGLELPETGG

HHHHHH

EGFP-LPETGG-His_6_

MASKGEELFTGVVPILVELDGDVNGHKFSVSGEGEGDATYGKLTLKFICT

TGKLPVPWPTLVTTLTYGVQCFSRYPDHMKRHDFFKSAMPEGYVQERTIF

FKDDGNYKTRAEVKFEGDTLVNRIELKGIDFKEDGNILGHKLEYNYNSHN

VYIMADKQKNGIKANFKIRHNIEDGSVQLADHYQQNTPIGDGPVLLPDNH

YLSTQSALSKDPNEKRDHMVLLEFVTAAGITHGMDELYKGSGLELPETGG

HHHHHH

DsRED-LPETGG-His_6_

MDNTEDVIKEFMQFKVRMEGSVNGHYFEIEGEGEGKPYEGTQTAKLQVTK

GGPLPFAWDILSPQFQYGSKAYVKHPADIPDYMKLSFPEGFTWERSMNFE

DGGVVEVQQDSSLQDGTFIYKVKFKGVNFPADGPVMQKKTAGWEPSTEKL

YPQDGVLKGEISHALKLKDGGHYTCDFKTVYKAKKPVQLPGNHYVDSKLD

ITNHNEDYTVVEQYEHAEARHSGSQLELPETGGHHHHHH

Tus-LPETGG-His_6_

MARYDLVDRLNTTFRQMEQELAIFAAHLEQHKLLVARVFSLPEVKKEDEH

NPLNRIEVKQHLGNDAQSLALRHFRHLFIQQQSENRSSKAAVRLPGVLCY

QVDNLSQAALVSHIQHINKLKTTFEHIVTVESELPTAARFEWVHRHLPGL

ITLNAYRTLTVLHDPATLRFGWANKHIIKNLHRDEVLAQLEKSLKSPRSV

APWTREEWQRKLEREYQDIAALPQNAKLKIKRPVKVQPIARVWYKGDQKQ

VQHACPTPLIALINRDNGAGVPDVGELLNYDADNVQHRYKPQAQPLRLII

PRLHLYVADGRGLELPETGGHHHHHH
