# Supplementary material for: Covalent Attachment of Proteins to Solid Supports and Surfaces via Sortase-Mediated Ligation
Source: PLoS One. 2007 Nov 14;2(11):e1164. doi: 10.1371/journal.pone.0001164 (PMC2063460; doi:10.1371/journal.pone.0001164)
Supplement: Data S2 — FACS data used to prepare Figure 1 and Figure 3. The mean fluorescence and standard error in the mean are given along with the number of events contributing to those values. (0.05 MB DOC) [file pone.0001164.s002.doc]

**Supplementary Data S2 – FACS data used in the preparation of Figures**

| Time (minutes) | 0 | 15 | 30 | 45 | 60 | 90 |
| --- | --- | --- | --- | --- | --- | --- |
| Tetraglycine beads | 225 ± 3  (891) | 2572 ± 21  (879) | 3083 ± 30  (877) | 3022 ± 19  (843) | 3343 ±28  (841) | 3637 ± 30  (879) |
| Diglycine beads | 150 ± 3  (858) | 2132 ± 27  (829) | 2494 ± 19  (861) | 2554 ± 21  (823) | 3056 ± 50  (872) | 3207 ± 21  (880) |
| Monoglycine beads | 189 ± 7  (260) | 720 ± 53  (79) | 728 ± 58  (75) | 1040 ± 75  (83) | 1222 ± 63  (93) | 1439 ±50  (176) |
| Amino beads | 127 ± 3  (869) | 138 ± 5  (370) | 142 ± 3  (787) | 150 ± 4  (781) | 162 ± 3  (791) | 230 ± 5  (436) |
| Negative control  (no Sortase) | 130 ± 3  (738) | 111 ± 4  (395) | 112 ± 3  (854) | 108 ± 3  (632) | 123 ± 2  (868) | 134 ± 4  (238) |

**Table 1. Data used in preparation of Figure 1**. Data reported are the mean fluorescence of the bead singlet population (see Methodology and Supplementary Figure S1) and the standard error in the mean of that population as reported by the FACSAria software. Fluorescence intensity is in arbitrary units and fluorescence and errors are rounded to the nearest whole number. The number of events contributing to the calculation of mean and standard error is given in brackets.

| [Ter DNA]  (Fluorescein label) | 0 | 20 | 40 | 60 | 80 | 100 |
| --- | --- | --- | --- | --- | --- | --- |
| [Non-specific DNA]  (Cy5 label) | 100 | 80 | 60 | 40 | 20 | 0 |
| Number of events | 4107 | 4255 | 4290 | 4323 | 2465 | 4328 |
| Fluorescein Fluorescence | 3940 ± 68 | 50199 ± 481 | 78133 ± 749 | 89996 ± 758 | 86391 ± 1139 | 102639 ± 935 |
| Cy5 Fluorescence | 94 ± 3 | 27 ± 1 | 29 ± 5 | 19 ± 4 | 0 ± 1 | 3 ± 1 |

**Table 2. FACS data used in preparation of Figure 3.** Data reported are the mean fluorescence of the bead singlet population (see Methodology) and the standard error in the mean as reported by the FACSAria software. Fluorescence intensity is in arbitrary units and fluorescence and errors are rounded to the nearest whole number.
