# Supplementary material for: Covalent Attachment of Proteins to Solid Supports and Surfaces via Sortase-Mediated Ligation
Source: PLoS One. 2007 Nov 14;2(11):e1164. doi: 10.1371/journal.pone.0001164 (PMC2063460; doi:10.1371/journal.pone.0001164)
Supplement: Figure S1 — Example raw FACS data for the labeling of diglycine modified GMA beads with EGFP-LPETGG-His6. A) Forward scatter versus side scatter (left panel). The central population (P1) consisting of single isolated beads is selected for further analysis. The values reported in Figure 1 are derived from analysis of this population. Forward scatter versus EGFP fluorescence (right panel) for diglycine beads after 30 minutes incubation with EGFP-LPETGG-His6 and Sortase. B) Histograms showing the increase in fluorescence over the course of the incubation. Times are as given at the top of each panel. The green population is the P2 selected in the the right hand panel of A). In all cases it is almost perfectly overlayed over the red P1 population selected on the basis of forward and side scatter, and from which average fluorescences were calculated. (0.04 MB DOC) [file pone.0001164.s003.doc]

**A**

**B**

**Supplementary Figure S1.** Example raw FACS data for the labeling of diglycine modified GMA beads with EGFP-LPETGG-His_6_. A) Forward scatter *versus* side scatter (left panel). The central population (P1) consisting of single isolated beads is selected for further analysis. The values reported in Figure 1 are derived from analysis of this population. Forward scatter *versus* EGFP fluorescence (right panel) for diglycine beads after 30 minutes incubation with EGFP-LPETGG-His_6_ and Sortase. B) Histograms showing the increase in fluorescence over the course of the incubation. Times are as given at the top of each panel. The green population is the P2 selected in the the right hand panel of A). In all cases it is almost perfectly overlayed over the red P1 population selected on the basis of forward and side scatter, and from which average fluorescences were calculated.
