# Supplementary material for: Covalent Attachment of Proteins to Solid Supports and Surfaces via Sortase-Mediated Ligation
Source: PLoS One. 2007 Nov 14;2(11):e1164. doi: 10.1371/journal.pone.0001164 (PMC2063460; doi:10.1371/journal.pone.0001164)
Supplement: Figure S2 — Original grey scale images used to construct Figure 2. Top panel show originals for Figure 2a and bottom panels show the original images used for preparation of Figure 2b. The dashed box shows the area of the original image that was expanded. Grey scale images were taken using the FITC or Cy3 filter sets on a Zeiss Axiovert 200 microscope using mercury vapour lamp illumination and captured on a Hamamatsu ORCA-ER camera using Simple PCI software. The exposure settings were selected using the ‘Auto Exposure’ setting of Simple PCI for each image. (1.53 MB DOC) [file pone.0001164.s004.doc]

Mixture of EGFP- and DsRed labelled GMA beads


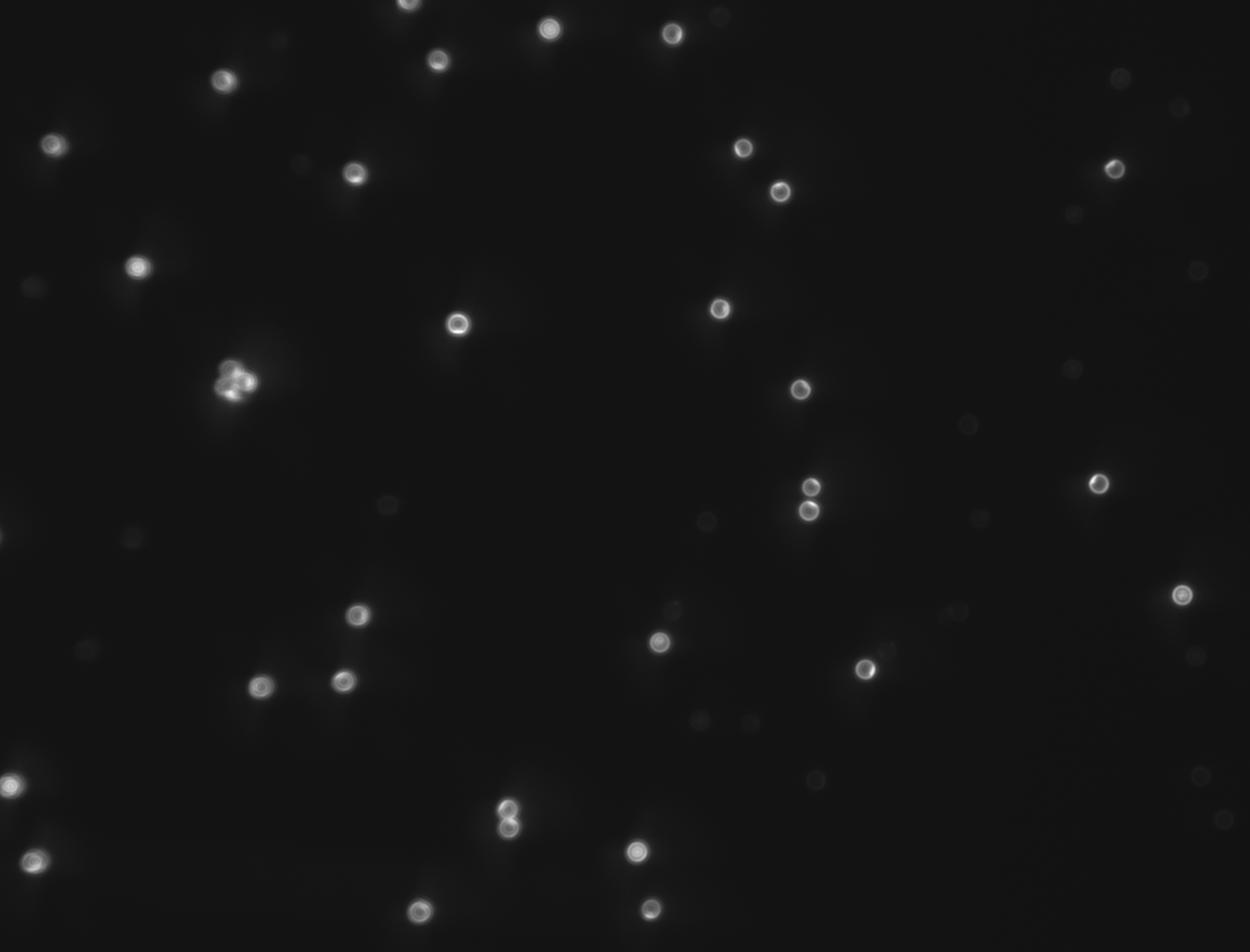

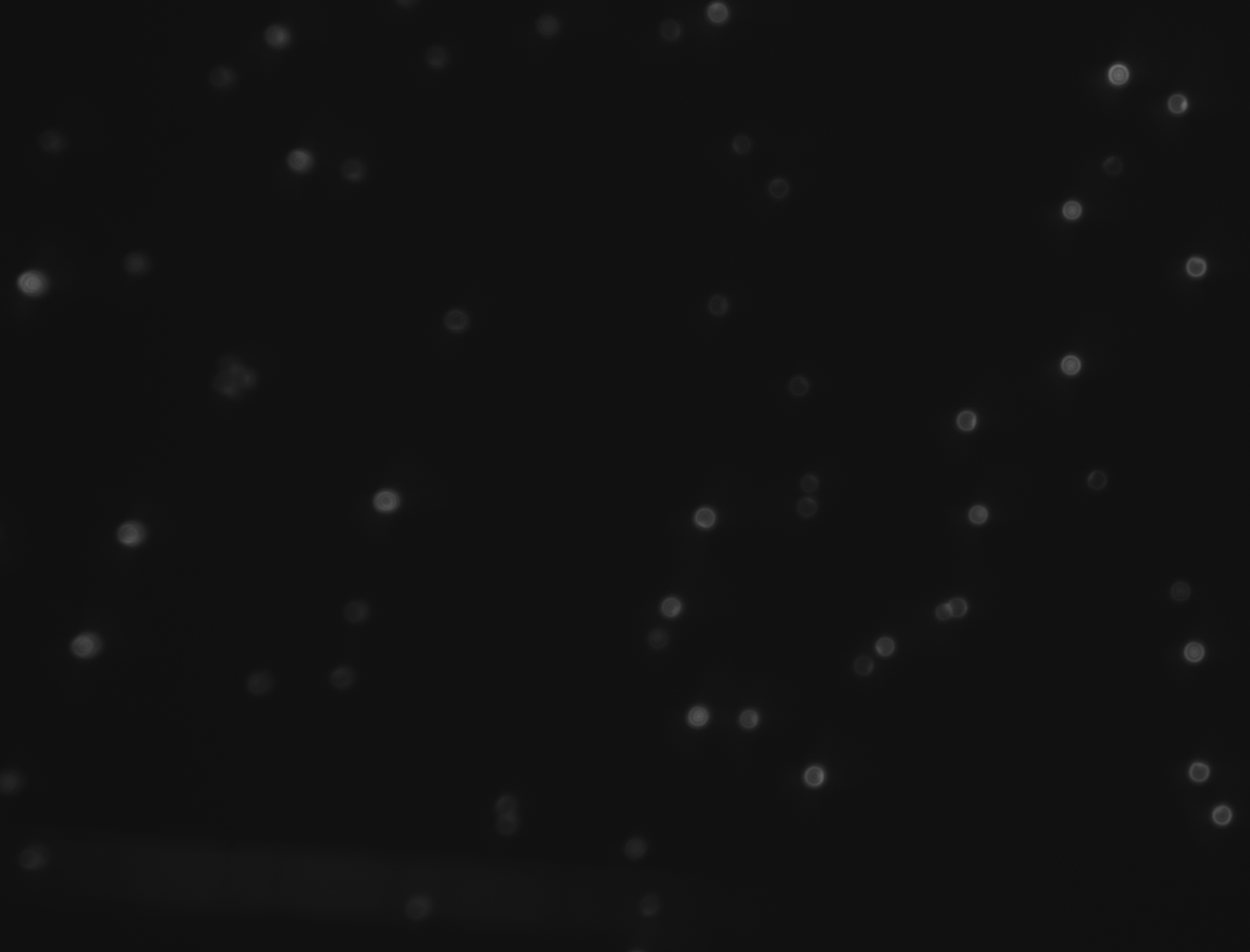


FITC filter set Cy3 Filter set

Mixture of EGFP- and DsRed labelled Affigel resin beads


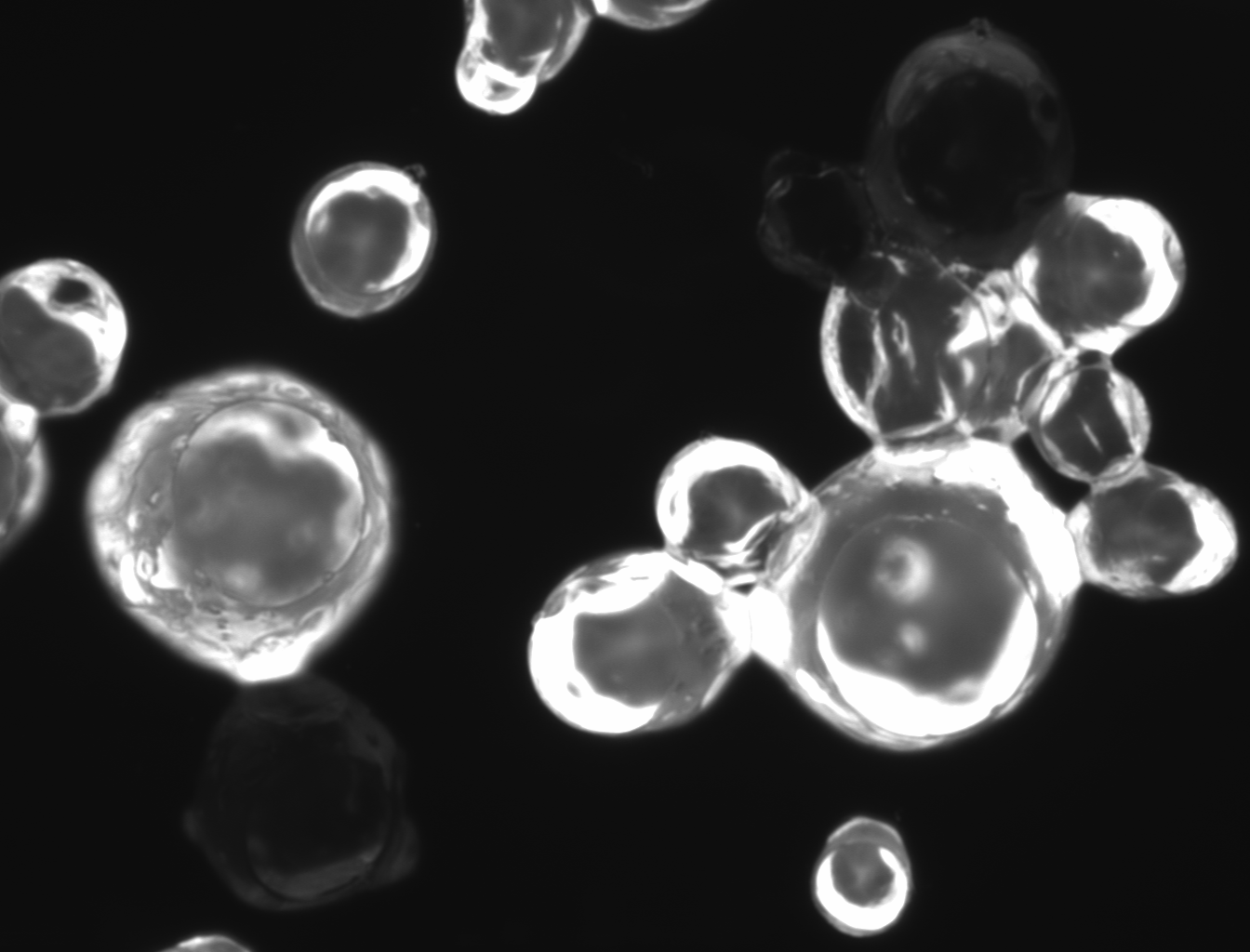

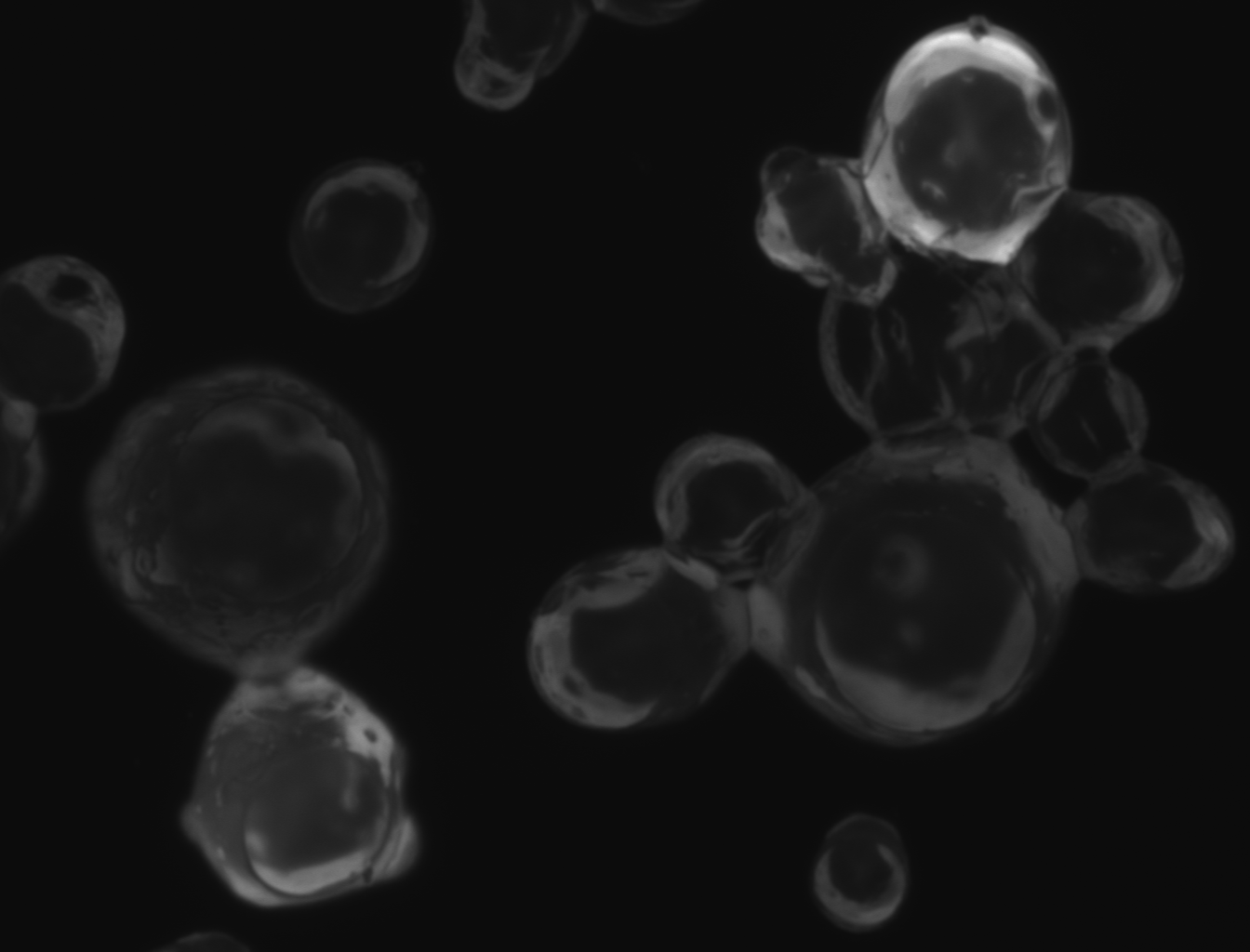


FITC filter Cy3 filter set

**Supplementary Figure S2.** Original grey scale images used to construct Figure 2. Top panel show originals for Figure 2a and bottom panels show the original images used for preparation of Figure 2b. The dashed box shows the area of the original image that was expanded. Grey scale images were taken using the FITC or Cy3 filter sets on a Zeiss Axiovert 200 microscope using mercury vapour lamp illumination and captured on a Hamamatsu ORCA-ER camera using Simple PCI software. The exposure settings were selected using the ‘Auto Exposure’ setting of Simple PCI for each image.
