# Supplementary material for: Covalent Attachment of Proteins to Solid Supports and Surfaces via Sortase-Mediated Ligation
Source: PLoS One. 2007 Nov 14;2(11):e1164. doi: 10.1371/journal.pone.0001164 (PMC2063460; doi:10.1371/journal.pone.0001164)
Supplement: Figure S3 — Unsuccessful preliminary attempts to immobilise EGFP on planar gold surfaces. The peptide H-GGC-OH (1 mM) was incubated overnight with a gold coated glass slide in the presence of triscarboxyethylphosphine (TCEP). The ligation of EGFP was attempted in the presence of 50 µM EGFP and 40 nM Sortase overnight. The successful immobilisation of the glycine peptide was not confirmed. Upper panel negative control (no Sortase). Lower panel, attempted ligation with Sortase. The lower panel is slightly out of focus. Similar results were seen with the peptides H-G3C-OH and H-G4C-OH. (0.53 MB DOC) [file pone.0001164.s005.doc]

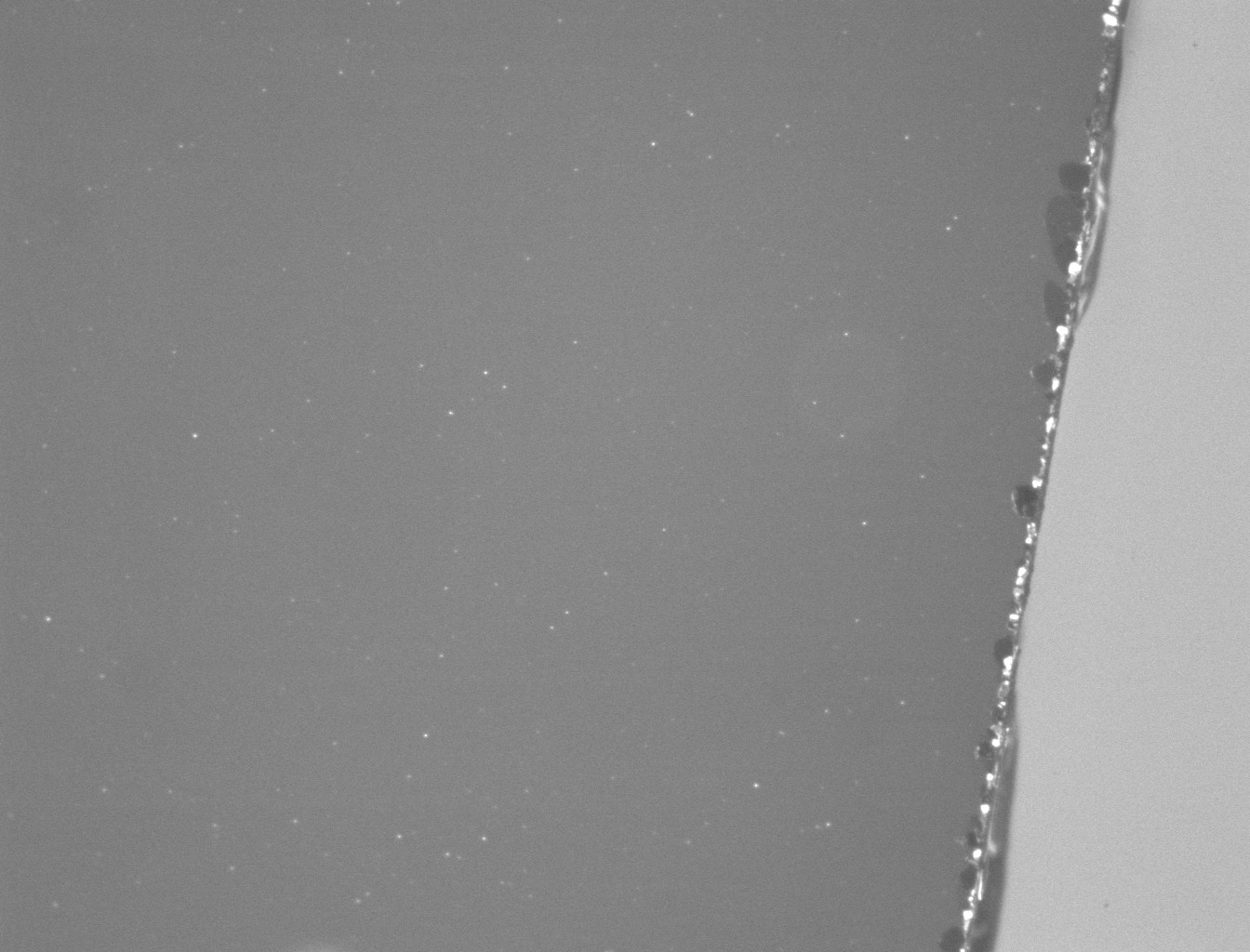


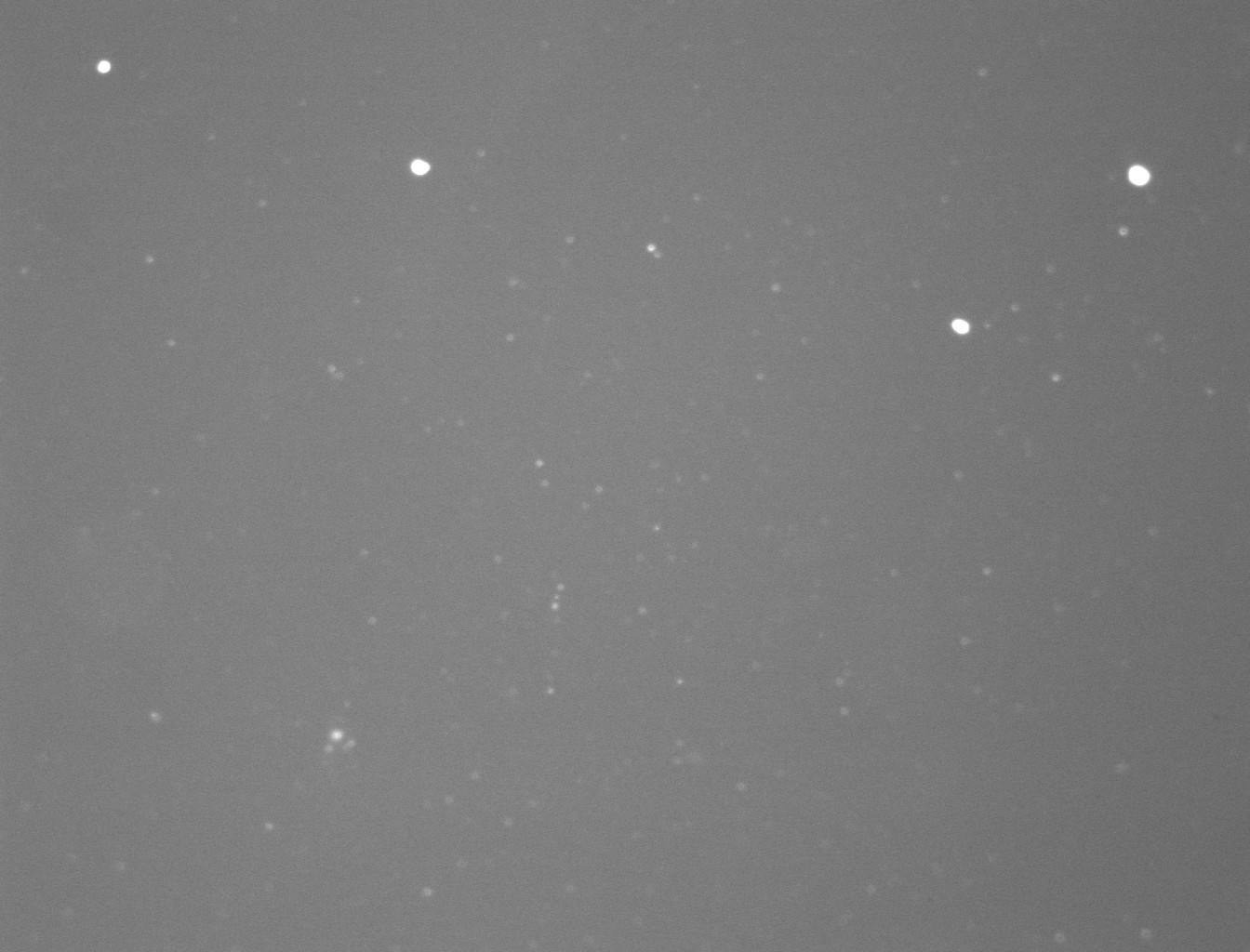


**Supplementary Figure S3.** Unsuccessful preliminary attempts to immobilise EGFP on planar gold surfaces. The peptide H-GGC-OH (1 mM) was incubated overnight with a gold coated glass slide in the presence of triscarboxyethylphosphine (TCEP). The ligation of EGFP was attempted in the presence of 50 μM EGFP and 40 nM Sortase overnight. The successful immobilisation of the glycine peptide was not confirmed. Upper panel negative control (no Sortase). Lower panel, attempted ligation with Sortase. The lower panel is slightly out of focus. Similar results were seen with the peptides H-G_3_C-OH and H-G_4_C-OH.
